# Effect of Oxidation on Quality of Chiba Tofu Produced by Soy Isolate Protein When Subjected to Storage

**DOI:** 10.3390/foods9121877

**Published:** 2020-12-17

**Authors:** Yue Xu, Zhongjiang Wang, Baokun Qi, Anqi Ran, Zengwang Guo, Lianzhou Jiang

**Affiliations:** College of Food Science, Northeast Agricultural University, Harbin 150030, China; xuyuename@neau.edu.cn (Y.X.); wzjname@neau.edu.cn (Z.W.); qibaokun@neau.edu.cn (B.Q.); anqiranraq@gmail.com (A.R.); gzwname@neau.edu.cn (Z.G.)

**Keywords:** Chiba tofu, soy protein isolate, storage, degree of oxidation, structural characteristics, rheology, texture, microstructure, sensory

## Abstract

Chiba tofu is a new type of vegetarian food prepared with soy protein isolate (SPI). According to factory feedback, the SPI stored in the factory storeroom in summer undergoes reactive oxidation, which changes the structure of SPI and further affects the quality of Chiba tofu. Consequently, the main objective of this study was to prepare Chiba tofu with SPI with different storage periods and evaluate the effect of different degrees of oxidation on structural characteristics of SPI and rheology, texture, microstructure and sensory properties of Chiba tofu. The carbonyl content and turbidity of SPI significantly increased, and the contents of free sulfhydryl (SH) and disulfide bond (S-S) simultaneously decreased with storage time. The oxidation changes the SPI conformation, leading to a transition of α-helix and β-turn to β-sheet and random coil during the storage periods. In the SDS–PAGE analysis, oxidation promoted the SPI molecules crosslinked and aggregated, which affected the quality of Chiba tofu. In short storage periods (0–12 days), SPI was relatively moderately oxidized when the carbonyl content was between 4.14 and 6.87 mmol/g. The storage and loss modulus of Chiba tofu both increased, the network was compact, and the hardness and springiness of Chiba tofu showed an increasing trend. Moreover, in longer storage periods (12–30 days), the SPI was relatively severely oxidized when the carbonyl content was between 7.24 and 9.14 mmol/g, which had an adverse effect on Chiba tofu rheological and texture properties, microstructure, and sensory properties. In sensory evaluation, Chiba tofu stored 12 days had the highest overall quality score than that stored on other days. This study is expected to provide an argument for the better industrial production of Chiba tofu.

## 1. Introduction

Soybeans are a kind of important legume that can be used to produce several soy products, including soy sauce, natto, tofu and fermented bean curd [1]. Among these, tofu is popular, with healthy nutritional properties and biological value [2]. The traditional tofu production process includes coagulating soymilk with calcium sulfate, magnesium chloride, or glucono δ-lactone (GDL), and then molding and pressing [3], which involves thermally induced gelation. However, the traditional tofu has weak gel strength, presumable due to denaturation of soybean protein under high-temperature, extensive crushing and shearing forces [3].

Chiba tofu is a new type of emergent vegetarian food. It is a kind of SPI gel, which involves cold gel formation induced by microbial transglutaminase (MTGase), and the addition of starch and salt to improve its flavor. MTGase is a coagulant of tofu-making [4], with some unique characteristics, for example, its improvement of retort-resistant and ease to control [5]. The cold gel overcomes the shortcomings of thermally induced gel methods and improves the gel strength of Chiba tofu [2]. Compared with traditional tofu, Chiba tofu gel strength is higher, and its taste is crisper, more elastic, and becomes increasingly popular in Asian countries.

SPI is a commercial plant protein and a functional ingredient in food processing with significant importance due to its good amino acid balance profit, emulsification ability, and strong gelation characteristic [6]. It is the main component of Chiba tofu, providing flavor and nutritional value. SPI is produced by defatted soybean flours. However, there is typically 1% residual lipids in the defatted soybean, which is easy to be catalyzed by lipoxygenase and further initiates lipid peroxidation (LPO). The reactive oxygen species (ROS), carbonyls and other reactive species, which are the intermediates of LPO, could cause the oxidation of protein [7]. Multiple structural changes are identified in oxidized proteins, including fragmentation of protein, protein-protein crosslinked polymers and interaction of proteins with other biomolecules during oxidation [8]. The gelling ability of protein changes as the extent of protein oxidation increases. Sochaya et al. [9] reported that severe oxidation led to an obvious reduction of gel strength and water-holding capacity of tilapia myosin. Zhou et al. [10] observed that moderate oxidation could improve in gel strength and network structure of myofibrillar proteins. Lu et al. [11] noted that mild protein oxidation could enhance the gel properties.

Moreover, due to the lack of equipment to stabilize protein structures and the distance between the SPI making plant and the Chiba tofu processing factory, SPI oxidation is inevitable, which affects the quality of Chiba tofu. According to the feedback of the factory working with our team, SPI storage in the factory storeroom over 30 days in summer is not commercially useful because it could not meet the requirements of downstream customers for quality standards, such as the taste and hardness of processing Chiba tofu. Therefore, we chose three batches of SPI as a repeated experiment, which were produced on August 1, August 2 and August 3, respectively. Each batch of SPI was stored for 30 days in the factory storeroom. Partly SPI was taken out when stored for 6, 12, 18, 24 and 30 days and made into Chiba tofu with the size of 3 × 3 × 3 cm^3^, aiming to (1) evaluate the oxidation degree of commercial SPI; (2) investigate the structure of commercial SPI affected by oxidation; (3) explore the changes in the quality of Chiba tofu over 30 days.

## 2. Materials and Methods

### 2.1. Materials

Three batches of SPI (protein content, 92% according to the manufacturer) were produced on August 1, August 2 and August 3, respectively, by the Lanshan Co. (Liaocheng, Shandong, China). Commercial MTGase, produced by *Streptomyces hygroscopicus*, was purchased from TAIXIN YIMIN Fine Chemical Industry Co. Ltd. (Nantong, Jiangsu, China). The enzyme activity of MTGase solution was 10 U/g as determined by a colorimetric procedure using Nα-CBZ-GLN-GLY as the substrate and L-glutamic acid γ-monohydroxamate as the standard, according to the method of Folk [12]. Soy oil, stretch and salt (COFCO Co. Ltd., Harbin, Heilongjiang, China) were purchased from a local market. All other chemicals were of analytical reagent grade and procured from Tianjin Chemical Reagent Co. (Tianjin, China).

### 2.2. Storage Experiment

SPIs produced on August 1, August 2 and August 3 were stored in Lanshan Co. Ltd. factory storeroom: sealed in dry ventilated place with room temperature below 30 °C and relative humidity below 65% On the 6th, 12th, 18th, 24th and 30th days of storage, part of the SPI was taken out as samples to make Chiba tofu.

### 2.3. Stepwise Method for Chiba Tofu Preparation

Chiba tofu with a size of 3 × 3 × 3 cm^3^ was prepared using three batches of SPI stored for 6, 12, 18, 24 and 30 days, following the method of Zheng et al. with slight modifications [13], and divided into 4 steps:

(A) Preparation of slurry: The SPI stock dispersion was prepared with SPI (15 g), starch (5 g) and distilled water (80 mL) in a Braun mixer (K600, Braun, Hamburg, Germany) at a speed setting of 3 (~1000 r/min) for 3 min. Then MTGase at an enzyme concentration of 10 U g^−1^ of protein was added in SPI dispersion and stirred for 2 min until homogeneous at a setting of 9 (~3000 r/min). Then, the mixture was mixed with soy oil, starch and salt and stirred at setting 3. Finally, setting 9 was used for 5 min under low-temperature using an ice bath to obtain a uniform slurry without small bubbles.
(B) Aging: The slurry was poured into a tray (26~36 cm) with a thickness of 3~4 cm. The slurry surface was covered with plastic wrap. Then, put the tray into the refrigerator (4 °C) for 10 h.
(C) Steam: the tray was placed on a water bath at 80–85 °C to float for 40 min until the center temperature was >75 °C.
(D) Pressing: After cooling down to room temperature, the samples were transferred to a plastic mold (3 × 3 × 3 cm^3^) and pressed with ion blocks. The pieces were put into a plastic food bag to store at (−18 ± 2 °C).

### 2.4. Carbonyl Analysis

The carbonyl content of protein was estimated by the method of Oliver et al. with slight modifications [14]. The samples of SPI at different storage periods were suspended in 10 mM sodium phosphate buffer (pH 7.0) and then stirred for 2 h. The 0.7 mL of solution was mixed with 2 mL of 10 mM 2,4 dinitrophenylhydrazine (DNPH), then incubated in the dark at 25 °C for 2 h. Moreover, an equal amount of sample was mixed with 3 mL of 2 M HCl as a blank. After incubating, the samples were mixed with 0.9 mL of 40% trichloroacetic acid (TAC). The mixtures were centrifuged at 10,000× *g* for 15 min at 4 °C, then washed three times with 3 mL of ethanol/ethyl acetate solution (1:1, *v*/*v*) to remove free DNPH. The final precipitate was dissolved in 1.2 mL of 6 M guanidine hydrochloride with 20 mM sodium phosphate buffer (pH 7.0). Absorbance at 367 nm was corrected by absorbance in the HCl blank. The results were calculated using a molar extinction coefficient of 22,000 M^−1^ cm^−1^ and expressed as nanomoles of carbonyl group per milligram of protein.

### 2.5. SH and S-S Content Analysis

The SH and S-S content of SPI were determined according to Ellman’s method [15]. Ellman’s reagent was prepared by dissolving 40 mg of 5,5′-dithiobis (2-nitrobenzoic acid) (DNTB) (Sigma Chemical Co., St. Louis, MO, USA) in 10 mL of methanol. The 75 mg of samples at different storage periods were solubilized in 10 mL of Tris-glycine buffer (0.086 M Tris, 0.09 M glycine and 4 mM ethylenediaminetetraacetic acid, pH 8.0) with 8 M urea. The solution was gently stirred overnight. For SH content determination, 4 mL of the Tris-glycine buffer was added to 1 mL of SPI solution. Then, 0.05 mL of Ellman’s reagent was added. The mixture was shaken and incubated at 25 °C for 24 h and then centrifuged at 20,000× *g* at 4 °C for 15 min. The supernatant fraction was analyzed for SH. The absorbance was measured at 412 nm on a UV-vis spectrophotometer (UV1100, Beijing Ruili Instrument Company Beijing, China).

For total SH content analysis, 4 mL of Tris-glycine buffer and 0.05 mL of 2-ME were mixed with 1 mL of the sample solution. The mixture was incubated with 10 mL of 12% TCA and centrifuged at 5000× *g* for 10 min. The precipitate was twice resuspended by 5 mL of 12% TCA and centrifuged to eliminate free 2-ME. The final precipitate was dissolved in 10 mL of Tris-glycine buffer. The 0.04 mL of Ellman’s reagent was mixed with 4 mL of the sample solution. The absorbance of the supernatant fraction was measured at 412 nm. The Tris-glycine buffer was used instead of SPI solutions as a reagent blank. A protein blank was used in which 0.04 mL of the Tris-glycine buffer replaced Ellman’s reagent solution. A reagent blank and sample blank were prepared to correct the color from reagents and protein solution. The calculation, according to the following Equation (1):

(1) μM SHg=73.53A412DC

where: *A*_412_ is the absorbance at 412 nm; *C* is the sample concentration in mg/mL; *D* is the dilution factor. The result was expressed as the mean of triplicate analysis.

Half of the value after subtracting the SH value from the total SH value was defined as the S-S content.

### 2.6. Turbidity Content Analysis

The 5 g of samples of SPI at different storage periods were solubilized 100 mL of deionized water at a concentration of 50 mg/mL and then stirred for 2 h at room temperature. The turbidity of the samples at different storage periods was measured with a UV spectrophotometer (TU 1900, Purkinje General Instrument Co., Beijing, China) at 600 nm. Turbidity was calculated by Equation (2):

(2) $$Turbidity=\frac{2.3.3\times A\times V}{L}$$

where: *A* is the absorbance of the samples at 600 nm, *V* is the dilution multiple (500), and *L* is the thickness of the samples (1 cm)

### 2.7. Raman Spectroscopy Analysis

The 10 g of samples of SPI at different storage periods were solubilized 100 mL of deionized water at a concentration of 100 mg/mL for Raman spectroscopy analysis. The Raman spectroscopy analysis was determined according to the method of Zhu et al. [16], using PerkinElmer Raman Station 400 F dispersive Raman spectrometer equipped with a 785 nm diode laser under 80 mW of laser power. The laser was focused on the samples on glass slides. For each sample, the spectrum measurement with 60 s exposure time was repeated 10 times scan for each sample under 2 cm^−1^ resolution, and the spectral range was set to 400~2000 cm^−1^. Each sample was scanned three times, and the average spectrum was used for a representative spectrum for each sample.

Quantitative analysis on the secondary structure of SP under specific conditions was performed by Gaussian fitting using the Peakfit 4.12 software (Seasolve Software, Framingham, MA, USA). Raman spectra (400–2000 cm^−1^) were plotted as relative intensity (arbitrary units) against Raman shift (wavenumber (cm^−1^)).

### 2.8. Sodium Dodecyl Sulfate-Polyacrylamide Gel Electrophoresis (SDS–PAGE) Analysis

The SDS-PAGE of samples at different storage periods were analyzedSDS–PAGE was carried out by a 12.5% separating gel and a 5% stacking gel. The 0.1 mL of sample was added to 0.9 mL of buffer containing 0.125 mol/L Tris-HCl, 2 g of SDS/100 mL, 5 mL of β-mercaptoethanol, 20 mL of bromophenol blue/100 mL, and heated at 95 °C for 5 min. The 7 µL of protein ladder and each sample were loaded into separate wells. After electrophoresis, the samples were stained with Coomassie blue R-250. The stained gels of the sample were digitized by the Epson Perfection 1270 image scanner (Epson America Inc., Long Beach, CA, USA) and analyzed by Gel-Pro Analyzer software (version 4.0, Media Cybernetics, Inc, Rockville, MD, USA).

### 2.9. Rheology Analysis

Cylinders of Chiba tofu (40 mm in diameter, 2 mm in thickness) were cut with a razor blade. The rheological properties of Chiba tofu were measured using a controlled-stress rheometer AR1000 (TA Instrument, New Castle, UK), which was equipped with parallel pate geometry (PP25, 25 mm diameter and 1 mm gap). The storage modulus (G′) and loss modulus (G′′) were recorded at a frequency sweep over a range of 0.1–10 Hz at 25 °C. All rheological measurements were carried out in triplicates.

### 2.10. Scanning Electron Microscopy (SEM) Analysis

The Chiba tofu was cut into a small piece (2 × 2 × 5 mm). Then, it was immersed in 2.5% glutaraldehyde and rinsed three times with phosphate buffer (pH 7.2). The rinsed slice was stepwise dehydrated using a serial ethanol concentration of 30%, 50%, 70%, 90% and 100%, and frozen in liquid nitrogen. The morphology of the lyophilized Chiba tofu was observed using a scanning electron microscope (JSM-6390 PLV, JEOL, Tokyo, Japan) at an accelerating voltage of 5 kV.

### 2.11. Texture Analysis

The texture profile analysis (TPA) of Chiba tofu was analyzed with a TA. XT, Plus Texture Analyzer (Stable Micro Systems, Goldaming, Surrey, UK) using a probe P26R, according to Noh et al. with some modifications [17]. The Chiba tofu was compressed twice with 70% compression at 25 °C, and the operating conditions of the apparatus were: speed 2.0 mm/s, probe diameter 25 mm, gels thickness 9 mm, trigger force 1 N. Three replicate tests were conducted for each sample.

### 2.12. Sensory Evaluations

Recruitment, selection, and training of panelists were performed according to sensory evaluation procedure [18]. Twelve panelists composed of students, faculty, and staff of the Northeast Agricultural University were randomly selected. They were trained with Chiba tofu for 2 weeks (three 30 min sessions per week) to familiarize the sample’s characteristics. A continuous linear-scale (15 cm) descriptive sensory characteristics evaluation method was used to evaluate the Chiba tofu in terms of firmness, elasticity, and smoothness. During the 2 weeks, they discussed and evaluated the marking positions (intensities) on the linear scale corresponding to characteristics of the Chiba tofu and until the panel reach consensus [19]. A marking position on the linear scale corresponding to the characteristic evaluation method was used to evaluate the characteristics of the Chiba tofu references. The commercial soft Chiba tofu reference (Liaocheng, Shandong, China) was designed at 2 cm for firmness and elasticity, respectively. The commercial firm Chiba tofu reference (Liaocheng, Shandong, China) was designed at 14 cm for firmness and elasticity, respectively. The commercial soft Chiba tofu reference was assigned at 12 cm for smoothness, whereas the firm Chiba tofu reference was assigned at 5 cm for smoothness. These assigned scores for the Chiba tofu reference were agreed upon by the panelists. Attributes of taste, flavor, color and overall quality tested were rated on a nine-point hedonic scale, where 9 stands for like extremely and 1 stand for dislike extremely [20]. During the evaluation of Chiba tofu samples, the panelist was not allowed to talk. Replicates of Chiba tofu samples (2 × 2 × 2 cm) in random code numbers were evaluated in different sessions and different days. Three tofu products were evaluated in each session.

### 2.13. Statistical Analysis

All determinations were performed at least in triplicate. The experimental data were subjected to one-way analysis of variance (ANOVA) using SPSS statistics software version 21 (IBM software, Armonk, NY, USA). Duncan’s multiple range tests were used to determine the statistical significance among the means at a 95% significant level. In this study, SPI of three batches and Chiba tofu prepared by three batches of SPI was used as the repeated experiment to confirm the repeatability and authenticity of the experiment. The results of the three repeated experiments were expressed as mean ± standard deviation.

## 3. Results

### 3.1. Determination of Oxidation Degree in SPI during the Storage Period

#### 3.1.1. Carbonyls Analysis

The carbonyl content in protein is reliable for oxidation because the protein carbonyls are formed as a result of oxidative modifications through the attack of ROS and its level indicates the extent of protein oxidation damage [21]. As present in Table 1, the carbonyl content of SPI increased with storage time, which indicates that the degree of oxidative damage increased. It was shown that SPI might react with peroxyl radical during storage, leading to the initial formation of a carbon-centered radical at either vulnerable amino acid side chains or backbone, and promote the carbonylation [22]. The loss of amino groups during protein carbonylation could alter electrical charges distribution, the isoelectric point of protein and the overall electrical arrangement of protein [23], which would break the balance between protein intermolecular and protein–water interactions and cause a favor of protein–protein interactions, eventually changing the protein structure [24]. Some of the protein peroxyl radicals are transformed to carbonyl derivatives in the subsequent reactions [25], and the latter, in turn, into crosslinks, leading to stretching and conformational changes [26].

The crosslinks of oxidized proteins have been demonstrated to possess the ability to form and stabilize the protein aggregates, which seriously affect protein structure and functionality [27]. The quality of Chiba tofu depends on the relationship between individual SPI, which is the main component of Chiba tofu. Therefore, the SPI structure changes caused by oxidative modifications during storage could cause negative effects in Chiba tofu.

#### 3.1.2. Disulfide Bond Analysis

The SH moiety on the side chain of the amino acid cysteine is able to interact with a variety of oxidants, to yield covalent modifications. In living systems, this modification is commonly used as an oxidation signal that may be transduced into a biological response [28]. These oxidations, as a mechanism by which protein function can be to control, include alteration of the redox state of cysteine in proteins and the sulfhydryl and disulfide exchange reaction of protein [29]. Protein sulfhydryl oxidation is divided into reversible form (sulfenic acid and protein disulfide) and irreversible form (sulfonic acid and sulfinic) in different oxidative environments [28]. The content of free SH and the S-S bond in SPI are shown in Table 1. The free SH group contents of SPI decreased from 1.86 to 1.40 mmol/g with storage time. The free SH groups may be oxidized. While the overall trend of the content of the S-S bond was downward with storage time. The contents of free SH and S-S simultaneously decreased, suggesting that the irreversible oxidation of SPI to form sulfur oxidation products rather than disulfide bonds.

#### 3.1.3. Turbidity Analysis

**Figure 1:**
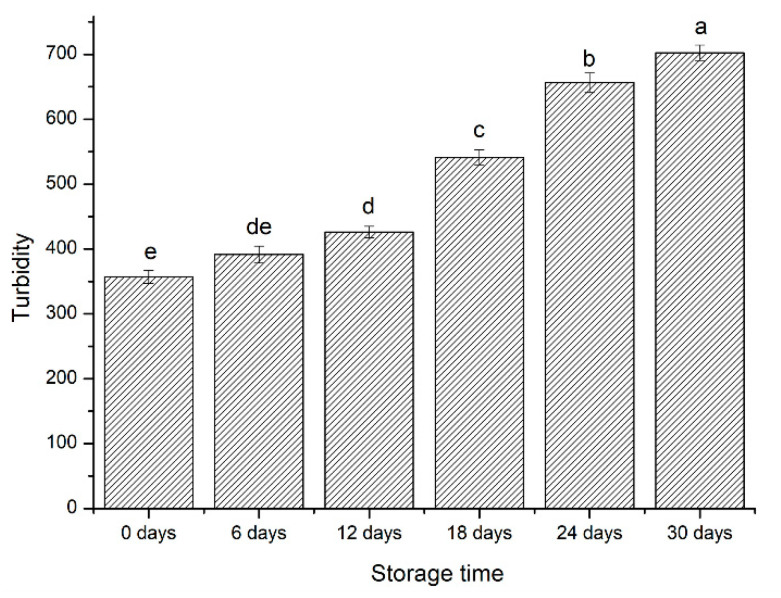
Turbidity of SPI (mmol∙mg^−1^) with different storage times. Different letters (a, b, c, d, e) indicate significant differences (*p* < 0.05).

Protein crosslinking could form protein aggregates, which is a complex phenomenon [30]. The protein solution turbidity is defined as the relative loss in the forward intensity of transmitted light [31], and it has become a convenient method to monitor protein aggregation because the turbidity is the function of the extent of light scattered by the aggregates [32]. Oxidative modification may attack amino acid side chains and the backbone of the protein and induce protein to unfold as well as to induce the exposure of hydrophobic groups. The protein aggregates are commonly formed by electrostatic interactions or hydrogen bonds [8]. The turbidity of SPI significantly increased with storage time (Figure 1), which was associated with increased oxidative damage.

### 3.2. Characterization of Structural Characteristics in SPI

#### 3.2.1. The Secondary Structure of SPI Analysis

**Figure 2:**
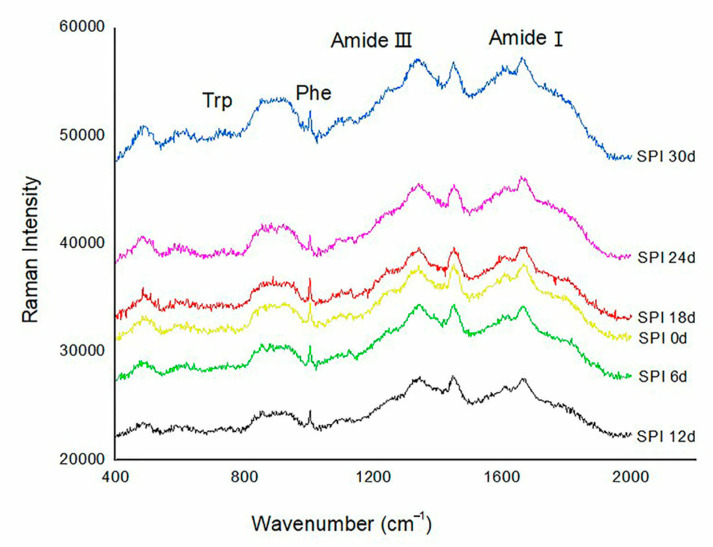
Raman spectra in the 400–2000 cm^−1^ region from SPI with different storage times.

The typical Raman spectra of SPI stored for different days in the 400–2000 cm^−1^ region are shown in Figure 2. Raman spectroscopy can provide information on the secondary structure of protein principally by examining the amide I region (1600–1700 cm^−1^), which is assigned to C=O stretching/hydrogen bonding coupled with COO- [16]. As showed in Figure 2, there was the maximum wavenumber in approximately 1650–1670 cm^−1^, which indicated that the α-helix conformation and random coil conformation were the primary conformations represent in SPI. The compositions of the secondary structure of SPIs at different storage periods are shown in Table 2. As listed in Table 2, the SPI exhibited 37.28% α-helix conformation, 11.99% β-sheet conformation, 15.18% turns conformation and 36.55% random coil conformation. The content of α-helix conformation and β-turn conformation decreased, whereas the content of β-sheet conformation and random coil conformation increased with storage time. The result indicated a conversion of SPI secondary structure from an ordered state to a disordered state during the storage period. The transition of α-helix and β-turn to β-sheet and random coil during the storage periods could be related to the formation of protein aggregates. Oxidative modification can induce partial protein to unfold and expose sites hidden within proteins. The aggregates were likely a result of hydrophobic interactions between the protein giving rise to the formation of constitute spherical protein aggregates through the β-sheet conformation [33]; hence the content of β-sheet would take place along with aggregation [34]. Additionally, the content of random coil conformation and β-turn conformation increased significantly, which was related to the aggregation behavior of soybean protein subunits, further indicating that SPI formed oxidative aggregation and the degree of protein molecular structure development was decreased [35]. Consistent with our findings, Sun et al. [36] also found that oxidation of myofibrillar proteins by hydroxyl radicals led to a decrease in α-helix conformation and β-turn conformation.

#### 3.2.2. SDS–PAGE Analysis

**Figure 3:**
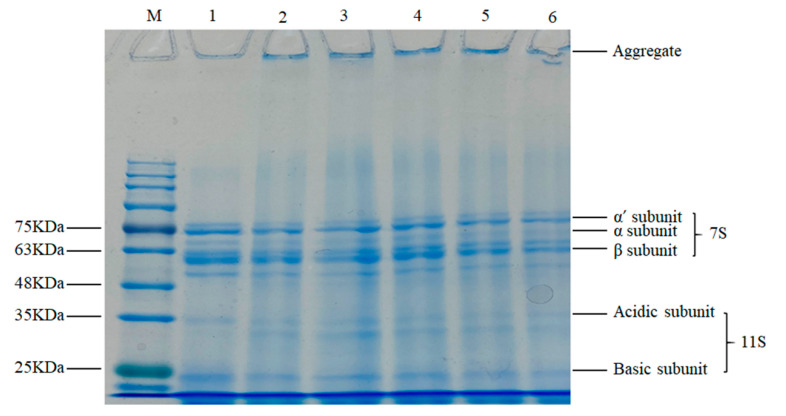
SDS–PAGE profile of SPI with different storage times.

The typical SDS–PAGE profile of SPIs are shown in Figure 3. The major components of SPI were β-conglycinin (7S) with α’ subunit (MW 83–57 kDa), α subunit (MW 76–57 kDa), β subunit (MW 53–42 kDa), and globulins glycinin (11S) with acidic subunit (MW 40–37 kDa) and basic subunit (19–20 kDa) [37]. According to Figure 3, the band intensities of SPIs gradually decreased with storage time, especially the α’ subunit, α subunit of 7S and the basic subunit of 11S bands were gradually lighter. It indicated that protein oxidation promoted aggregation between the subunits of 7S and 11S. Meanwhile, the long-term storage resulted in the formation of insoluble crosslinking high molecular of protein aggregate (Figure 3), which prevented them from entering the electrophoretic gel [37]. Kong and Chang [38] already observed that a consequence of soy protein storage is the formation of extensive protein aggregates, which could not penetrate into the acrylamide gel.

### 3.3. Characterization of Quality of Chiba Tofu

#### 3.3.1. Rheological Analysis

**Figure 4:**
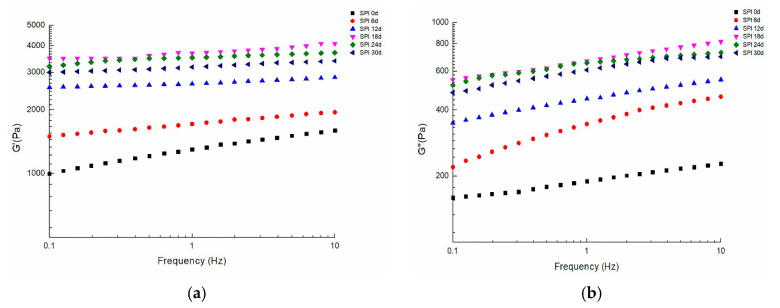
Frequency sweep the G′ (**a**) and G′′ (**b**) of Chiba tofu with different storage times.

**Figure 5:**
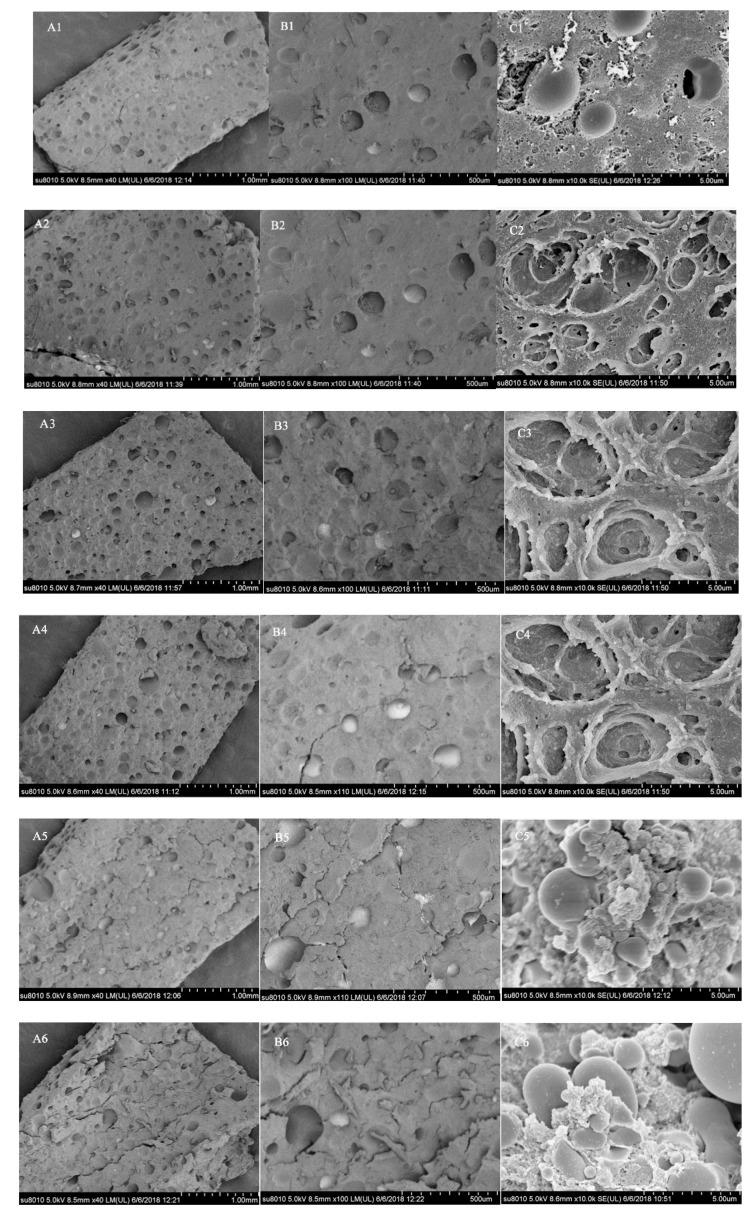
Scanning electron microscope of the Chiba tofu. Numbers 1, 2, 3, 4, 5 and 6 represent the samples of SPI that were stored for 0 days, 6 days, 12 days, 18 days, 24 days and 30 days, respectively. (**A1**–**A6**,**B1**–**B6**,**C1**–**C6**) represent the magnification of 40, 100 and 10,000.

The typical frequency sweep plots for Chiba tofu in different storage periods are shown in Figure 4. The G′ was higher than G′′, indicating that there was a continuous network structure in Chiba tofu. It has been reported that the faster molecular mobility of solid particles was connected with the frequency-dependent property of G′ and G″ [39]. Both G′ and G′′ slightly increased as the increasing frequency of oscillating stress, which demonstrated that the characteristic of the Chiba tofu prepared in this study was close to a viscoelastic material [40]. The G′ and G′′ first increased and latterly slightly decreased with storage periods. As a result of oxidation, soluble protein aggregates during storage, and this could result in a great extent of crosslinks and lead to thicker strands and denser network structures with higher storage and loss moduli [41]. However, severe oxidation in further stages resulted in the formation of a discontinuous network of Chiba tofu (Figure 5) and therefore had an adverse effect on the quality of Chiba tofu.

#### 3.3.2. SEM Analysis

The SEM of Chiba tofu prepared by SPI stored at different times is shown in Figure 5. SEM micrograph of Chiba tofu, which was similar to other tofu, had an opaque white appearance [29]. SEM image of the Chiba tofu without storage periods showed a compact and evenly distributed network with a lesser number of interspaces (Figure 5(A1–C1)). When the SPIs were stored for 6 and 12 days, the Chiba tofu exhibited a honeycomb structure with greater interspaces of protein strands (Figure 5(C2,C3)), which could be attributed to the promotion of the denaturation and unfolding of protein under moderate oxidation [42], meanwhile, the hardness and springiness of the Chiba tofu improved (Table 3). Noh [17] also reported that the more orderly and denser is the structure, the higher are the values of textural parameters. When the SPIs were stored for 18, 24 and 30 days, the Chiba tofu showed network ruptured network under the influence of oxidative modification (Figure 5(B4–B6)), and there are some aggregated matrixes with large cavities in Chiba tofu. (Figure 5(C4–C6). The result shows that oxidative protein aggregation will destroy the stability of the SPI structure and reduce the strength of the gel, changing its microstructure [43]. Wang [44] also pointed out that the protein gel ordered network structure was remarkably affected by oxidation and showed a formation of large aggregates.

#### 3.3.3. Texture Analysis

The hardness, cohesiveness, springiness, gumminess, and chewiness of Chiba tofu are shown in Table 3. The hardness increased, gumminess and chewiness gradually decreased, and the springiness increased first and then decreased with a storage time of SPI, while no significant difference was observed in the cohesiveness of Chiba tofu. The oxidative damage of SPI could lead to aggregation and complex formation via crosslinking [8], which could increase the hardness and springiness of Chiba tofu. However, one of the main consequences of severe crosslinks and aggregation in oxidized protein is obstruction interactions between reactive functional groups, and in some cases, also reduction of hydrogen bonds in the protein matrix. The association of protein–protein and protein–water could be a remarkable influence [45], eventually resulting in a decrease in springiness. Kamizake [46] also pointed out that the protein aggregation would change the protein molecular structure, causing a failed ability to form a suitable gel network. The change of Chiba tofu texture was similar to the change of Chiba tofu microstructures.

#### 3.3.4. Sensory Evaluation

Sensory evaluation was conducted twice for each Chiba tofu in different periods to test the quality (including for firmness, elasticity, smoothness, taste, flavor and color) (Table 4). The sensory result showed a statistically significant difference among Chiba tofu after steaming in different storage periods in firmness, elasticity, smoothness, taste and flavor, and the non-significant difference in color. Meanwhile, the sensory result of Chiba tofu stored for 6 and 12 days was improved. For the firmness and elasticity, all Chiba tofu varied from 3.1 to 9.7 and from 4.7 to 7.5, respectively. With the prolongation of the storage period, the Chiba tofu showed lower firmness and elasticity. They were consistent with the results of texture properties. There was no significant difference in the smoothness of Chiba tofu. The sensory smoothness of Chiba tofu in different storage periods varied from 7.6 to 9.9. Compared with commercial soft Chiba tofu score of 12, the samples tofu showed a lower smoothness but was of higher smoothness (score: 5). For the taste and flavor, all Chiba tofu varied from 4.0 to 6.1 and 4.4 to 7.2, respectively. The combined effect of Chiba tofu sensory parameter scores was recorded as overall quality. Chiba tofu stored for 30 days was lower than the other samples. Chiba tofu stored 12 days had the highest overall quality score than that stored for a different period. A significant positive correlation was observed between overall quality and taste as well as flavor.

## 4. Conclusions

The degree of oxidation changes SPI structural characteristics during storage time. In carbonyl, sulfhydryl group and turbidity content analysis, we found the SPI underwent an irreversible oxidation reaction and simultaneously, the degree of oxidation and protein aggregation increased with storage time. The content of α-helix conformation and β-turn conformation decreased, whereas the content of β-sheet conformation and random coil conformation increased. Meanwhile, the oxidation promoted aggregation between the subunits of 7S and 11S and resulted in the presence of a high molecular weight region under SDS–PAGE with the storage periods. In the early storage periods (0–12 days), the G′ and G′′ of Chiba tofu both increased, the network was denser, hardness and springiness were increased. Moreover, in the later storage periods (12–30 days), the severe oxidation had an adverse effect on Chiba tofu characterization, which reflected in a rupture of network structure with large cavities and decrease in springiness of Chiba tofu. In sensory evaluation, Chiba tofu stored 12 days had the highest overall quality score than that stored on other days. Above all, the quality of Chiba tofu was better in the first 12 days, which provided a theoretical basis for the industrial production of soy products.

## Figures and Tables

**Table 1:** Protein oxidation markers in soy protein isolate (SPI) with different storage times.

Storage Time (Day)	Protein Carbonyl (mmol/g)	Free SH (mmol/g)	S-S Content (mmol/g)
0	4.14 ± 0.15 ^f^	1.86 ± 0.01 ^a^	1.31 ± 0.02 ^b^
6	5.25 ± 0.09 ^e^	1.83 ± 0.03 ^b^	1.79 ± 0.02 ^a^
12	6.87 ± 0.17 ^d^	1.63 ± 0.03 ^c^	1.25 ± 0.10 ^b^
18	7.24 ± 0.19 ^c^	1.51 ±.0.01 ^d^	1.12 ± 0.10 ^c^
24	8.17 ± 0.17 ^b^	1.41 ± 0.03 ^f^	0.89 ± 0.01 ^d^
30	9.14 ± 0.11 ^a^	1.40 ± 0.01 ^e^	0.79 ± 0.05 ^d^

Different letters (^a, b, c, d, e, f^) in the same column indicate significant differences (*p* < 0.05).

**Table 2:** The relative content of secondary structures of SPI with different storage times.

Storage Time (day)	The Relative Content of Secondary Structures (%)			
	α-Helix	β-Sheet	β-Turn	Random Coil
0	37.28 ± 0.14 ^a^	11.99 ± 0.12 ^e^	15.18 ± 0.11 ^a^	36.55 ± 0.14 ^f^
6	36.06 ± 0.15 ^b^	12.02 ± 0.15 ^de^	14.87 ± 0.12 ^b^	37.03 ± 0.17 ^e^
12	36.91 ± 0.20 ^b^	12.30 ± 0.13 ^d^	14.32 ± 0.18 ^c^	37.47 ± 0.15 ^d^
18	35.25 ± 0.12 ^c^	13.41 ± 0.17 ^c^	13.35 ± 0.15 ^d^	37.99 ± 0.08 ^c^
24	34.07 ± 0.02 ^d^	14.56 ± 0.06 ^b^	12.37 ± 0.20 ^e^	38.97 ± 0.11 ^b^
30	33.65 ± 0.05 ^d^	15.27 ± 0.16 ^a^	11.61 ± 0.14 ^f^	39.47 ± 0.11 ^a^

Different letters (^a, b, c, d, e, f^) in the same column indicate significant differences (*p* < 0.05).

**Table 3:** Texture profile analysis of Chiba tofu gels with different storage times.

Storage Time (day)	Hardness (g)	Cohesiveness	Springiness (mm)	Gumminess (g)	Chewiness (g mm)
0	223.64 ± 1.81 ^f^	0.93 ± 0.01 ^ab^	1.01 ± 0.01 ^c^	309.08 ± 0.88 ^a^	340.57 ± 0.02 ^a^
6	234.61 ± 1.48 ^d^	0.92 ± 0.01 ^b^	1.07 ± 0.02 ^b^	286.81 ± 0.61 ^b^	287.77 ± 0.02 ^b^
12	237.81 ± 1.55 ^e^	0.94 ± 0.01 ^ab^	1.13 ± 0.01 ^a^	239.84 ± 0.87 ^c^	230.36 ± 0.02 ^c^
18	257.20 ± 2.01 ^c^	0.93 ± 0.01 ^ab^	1.09 ± 0.01 ^b^	221.17 ± 0.75 ^d^	219.38 ± 0.03 ^d^
24	292.84 ± 1.33 ^b^	0.94 ± 0.02 ^a^	0.99 ± 0.01 ^d^	214.81 ± 0.43 ^e^	222.56 ± 0.06 ^e^
30	329.05 ± 0.44 ^a^	0.93 ± 0.01 ^ab^	0.96 ± 0.01 ^d^	203.87 ± 0.95 ^f^	202.85 ± 0.04 ^f^

Different letters (^a, b, c, d, e, f^) in the same column indicate significant differences (*p* < 0.05).

**Table 4:** Sensory evaluation of Chiba tofu with different storage times.

Storage Time (day)	Continuous Linear-Scale			Nine-Point Hedonic Scale			
	Firmness	Elasticity	Smoothness	Taste	Flavor	Color	Overall, Quality
0	3.1 ± 0.12 ^d^	4.7 ± 0.42 ^f^	7.6 ± 0.53 ^b^	6.1 ± 0.02 ^a^	7.2 ± 0.02 ^a^	7.0 ± 0.14 ^a^	6.9 ± 0.09 ^c^
6	3.7 ± 0.23 ^d^	5.1 ± 1.20 ^e^	7.5 ± 1.01 ^b^	6.6 ± 0.04 ^b^	6.9 ± 0.05 ^b^	7.0 ± 0.16 ^a^	7.8 ± 0.07 ^b^
12	5.4 ± 0.74 ^d^	7.5 ± 0.78 ^a^	7.9 ± 1.15 ^b^	7.9 ± 0.01 ^c^	6.7 ± 0.02 ^c^	6.9 ± 0.13 ^a^	8.2 ± 0.05 ^a^
18	6.3 ± 0.24 ^c^	7.2 ± 0.17 ^b^	8.5 ± 0.90 ^ab^	5.7 ± 0.06 ^d^	6.5 ± 0.01 ^d^	6.9 ± 0.16 ^a^	6.5 ± 0.01 ^d^
24	7.5 ± 0.79 ^b^	6.9 ± 0.87 ^c^	9.7 ± 1.12 ^a^	4.9 ± 0.03 ^e^	5.7 ± 0.04 ^e^	7.0 ± 0.11 ^a^	6.1 ± 0.04 ^e^
30	9.7 ± 1.03 ^a^	6.3 ± 1.21 ^d^	9.9 ± 0.85 ^a^	4.0 ± 0.03 ^f^	4.4 ± 0.01 ^f^	6.9 ± 0.15 ^a^	5.2 ± 0.03 ^f^
Soft Chiba tofu	2	2	12	-	-	-	-
Firm Chiba tofu	14	14	5	-	-	-	-

All tests were conducted twice on different days for the tofu made from the same line, using the commercial tofu (soft Chiba tofu and firm Chiba tofu shown in the last two rows) as references. Different letters (^a, b, c, d, e, f^) in the same column indicate significant differences (*p* < 0.05).
